# Job Strain, Health and Sickness Absence: Results from the Hordaland Health Study

**DOI:** 10.1371/journal.pone.0096025

**Published:** 2014-04-22

**Authors:** Min-Jung Wang, Arnstein Mykletun, Ellen Ihlen Møyner, Simon Øverland, Max Henderson, Stephen Stansfeld, Matthew Hotopf, Samuel B. Harvey

**Affiliations:** 1 School of Psychiatry, University of New South Wales, Sydney, Australia; 2 Department of Health Promotion and Development, Faculty of Psychology, University of Bergen, Bergen, Norway; 3 Department of Public Mental Health, Division of Mental Health, Norwegian Institute of Public Health, Bergen, Norway; 4 Department of Psychological Medicine, Institute of Psychiatry, King’s College London, London, United Kingdom; 5 Centre for Psychiatry, Wolfson Institute of Preventive Medicine, Queen Mary University of London, London, United Kingdom; 6 Black Dog Institute, Sydney, Australia; McGill University, Canada

## Abstract

**Objectives:**

While it is generally accepted that high job strain is associated with adverse occupational outcomes, the nature of this relationship and the causal pathways involved are not well elucidated. We aimed to assess the association between job strain and long-term sickness absence (LTSA), and investigate whether any associations could be explained by validated health measures.

**Methods:**

Data from participants (n = 7346) of the Hordaland Health Study (HUSK), aged 40–47 at baseline, were analyzed using multivariate Cox regression to evaluate the association between job strain and LTSA over one year. Further analyses examined whether mental and physical health mediated any association between job strain and sickness absence.

**Results:**

A positive association was found between job strain and risk of a LTSA episode, even controlling for confounding factors (HR = 1.64 (1.36–1.98); high job strain exposure accounted for a small proportion of LTSA episodes (population attributable risk 0.068). Further adjustments for physical health and mental health individually attenuated, but could not fully explain the association. In the fully adjusted model, the association between high job strain and LTSA remained significant (HR = 1.30 (1.07–1.59)).

**Conclusion:**

High job strain increases the risk of LTSA. While our results suggest that one in 15 cases of LTSA could be avoided if high job strain were eliminated, we also provide evidence against simplistic causal models. The impact of job strain on future LTSA could not be fully explained by impaired health at baseline, which suggests that factors besides ill health are important in explaining the link between job strain and sickness absence.

## Background

Long-term sickness absence (LTSA) has become a major public health problem in most developed countries [1]. Self-report surveys, such as the UK’s Labor Force Survey, suggest that stress is now the leading ‘cause’ of work-related illnesses, accounting for around 40% of all new incidences of SA episodes [2], leading some to describe work-related stress as a “modern epidemic” [3]. Currently, the most dominant model of job stress is Karasek’s demand-control model [4], which is comprised of two main components: self-perceived job control and psychological demands. Job control is characterized by decision authority and skill discretion, while psychological demand is a function of workload, conflicting demands and work pressure. The job strain hypothesis is derived from this model and suggests that psychological demands will have the most negative impact on well-being in the setting of low job control. As such, high strain jobs are those that combine high demands with low control.

While job strain has been linked to increased rates of mental illness and cardiovascular disease [5], [6], a recent review concluded that the causal association between job strain and negative occupational outcomes, such as LTSA, remains equivocal [7]. Although there is reasonable evidence demonstrating the negative association between job control and sickness absence [8], [9], findings for job demand have been inconclusive [10], [11]. Studies assessing the combined effects of job demand and control also produced inconsistent results. While some studies demonstrated significant associations between job strain and sickness absence [12], [13], other studies did not [14]. These discrepancies may be because few studies have accounted for sickness absence at baseline, which may lead to an overestimation of the strength of association. Furthermore, many studies have been cross-sectional, often restricted to male populations, based on unofficial records of sick leave and have not sufficiently accounted for confounders.

While most researchers in the field of occupational medicine have always been aware of the complex nature of any links between job strain and sickness absence, the practical interpretation of the job strain literature has, at times, led some to assume that there is a simple causal chain, with job strain leading to poorer health, which then leads to sickness absence [15]. However, there is increasing evidence that there is a range of other non-health factors which may predict long term sickness absence [16]–[18], which has contributed to the long held understanding that other pathways may affect the relationship between job strain, ill health, and thus sickness absence [15], [19]. Researchers have postulated that both the assessment and effects of job strain could be influenced by individual factors, including job satisfaction [9], education [16], and personality characteristics [20]. These factors may be vital in explaining the apparent association between self-reported job strain and long term sickness absence. In addition, individual thresholds and perceptions of vulnerability [21], lifestyle [22] and attitudes towards work [15] may also influence both the assessment of job strain and the likelihood for taking sick leave once symptoms are present. Furthermore, prior studies have shown that factors such as gender, occupational class [23] and family demands [24] may modify the effects of psychosocial work factors on sickness absence. As a result, any link between job strain, ill health and sickness absence is unlikely to be simple [15]. A better understanding of how various individual, health and job factors combine to predict sickness absence behavior is vital in order to assist the development of effective rehabilitation strategies aimed at reducing long term disability.

The main objective of this study was to determine whether high job strain is associated with increased risk of long-term SA (>16 days), using health data linked to official Norwegian records, over a 1-year follow up. We further aimed to investigate whether the association could be explained by impairments in both physical and mental health.

## Materials and Methods

### Hordaland Health Study (HUSK)

The Hordaland Health Study (HUSK) was an epidemiological population-based health survey (1997–1999) carried out by the Norwegian Health Screening Service in collaboration with the University of Bergen, Norway. The base population included 29,400 individuals aged 40–47 living in Hordaland county. 18,581 individuals completed the first questionnaire and the clinical examinations (participation rate 63%). 50% of these men and 75% of these women were randomly selected to fill out a second questionnaire which included the Swedish Demand-Control-Support Questionnaire (DCSQ) (the motivation for the oversampling of women was related to other questionnaires contained within this section).

### Ethics Statement

The study protocol for the HUSK Study was approved by the Regional Ethics Committee of Western Norway and the Norwegian Data Inspectorate. All participants provided written informed consent for participation in the study at baseline.

### Study Sample

The base population included individuals with valid responses to the second questionnaire (n = 8896). We excluded individuals who were not in paid employment or were already taking sickness-related absence at baseline. We further removed individuals who were receiving disability pensions or were awarded with disability pension benefits within 13 months of baseline, as these were likely to have uncertain relationships with work and health. Finally, those without valid DCSQ scores were removed from analysis; the final study sample consisted of 7346 individuals.

### Job Strain

Workplace psychological demands and job control were measured using the 17-item Swedish Demand-Control-Support Questionnaire (DCSQ) [25] developed by Karasek and Theorell [26]. The demand subscale has five items that measures work pace and occurrence of conflicting demands. The control/decision latitude subscale includes six items; two measuring decision authority and four measuring skill discretion. Due to translation error from Swedish to Norwegian, one decision latitude item was excluded, but the 16-item Norwegian version of the DCSQ has satisfactory psychometric properties [25].

The variables representing psychological demands and job control were dichotomized at the median of the study population’s score distribution. This produced four categories of exposure: low strain (low demands with high control), active work (high demands with high control), passive work (low demands and low control) and high strain (high demands and low control). For the analyses, active work and passive work were grouped together as intermediate strain, as previous studies have shown that the two groups did not differ on the Job Content Survey (JCS) strain scale [27].

### Sickness Absence

Information on medically verified sickness absence (SA) awarded until the end of 2003 was accessed through official Norwegian registries of state paid SA benefits (FD-trygd) and linked with HUSK data through Statistics Norway. The SA records are highly accurate as correct registration is required for the transfer of payments by the social insurance scheme. In Norway, employers are responsible for covering the first 16 calendar days of SA on the condition that the employee had worked for 4 weeks prior to the SA episode. Beyond this period, the National Insurance Scheme pays for absences of up to 52 weeks. There is no universally accepted definition of long-term sickness absence (LTSA) [28]; in this study, LTSA was defined as absence from work for more than16 days.

### Baseline Characteristics

Information on age and gender was obtained through the Norwegian Population Register. The HUSK questionnaire provided data on the highest education level, marital status, income after tax, and these were used as indicators of socioeconomic status (SES) - a method employed by a previous study also based on HUSK data [29]. In addition, the number of biological children that each participant had was also available. Lifestyle characteristics including physical activity, smoking habits and alcohol consumption, were also collected. Body mass index (BMI, kg/m^2^) was calculated using height and weight measurements.

### Mental and Physical Health

Common mental disorder symptoms were evaluated through the Hospital Anxiety and Depression Scale (HADS) [30]. HADS consists of two subscales that measure symptoms of anxiety (HADS-A) and depression (HADS-D). Self-perceived mental health was measured using the mental composite score (MCS), a subscale of the Short Form-12 (SF-12) Health Survey [31].

Physical health was measured using four different indicators; chronic somatic diseases, pharmacological diagnoses, somatic symptoms, and self-perceived physical health status. Chronic somatic diseases were assessed by self-reported occurrences of myocardial infarction, stroke, diabetes, asthma, angina pectoris, diabetes or multiple scleroses; from these, a continuous variable (ranging 0–6) was created [32]. The pharmacological diagnoses variable was represented by the number of somatic symptoms under pharmacological treatment, which was based on medications taken by participants at baseline [33]; a panel of physicians assigned appropriate diagnoses for the likely physical condition based on the International Classification of Primary Care diagnoses according to the Anatomical Therapeutic Classifications System. The variable for somatic symptoms indicated the frequency of experiencing common somatic symptoms in accordance with the ICD-10 research criteria for F45-Somatoform Disorders [34]. Self-perceived physical health status was assessed using the physical composite score (PCS) subscale of the SF-12.

### Statistical Analysis

For baseline characteristics, we ran descriptive statistics and also investigated their univariate associations with LTSA. Participants were censored after their first LTSA episode or if they died during the follow-up period before an episode. Mental health and physical health were identified *a priori* as possible mediators of the relationship between job strain and LTSA; therefore we assessed their associations with both job strain and LTSA. Mental and physical health were compared across job strain categories using the Kruskal Wallis test for continuous variables, and the chi-squared or Fisher’s exact test for categorical variables. The associations between the standardized health measures (z-scores) and sickness absence were analyzed using univariate Cox regression models.

Multivariate analysis, adjusted for potential confounders and mediators separately, was employed to study the association between job strain and sickness absence. The results are presented as hazard ratios (HRs) with 95% confidence intervals (95% CI). To investigate the possibility of gender, age, and number of biological children as effect modifiers of this association [35], [36], multiplicative interaction terms were included in the regression models. The population attributable fraction (PAF) [37] was also computed, representing the percentage of LTSA episodes that could have been prevented had the study population not been exposed to high job strain. All statistical analyses were conducted using STATA v.12.0 [38].

## Results

Of the 7346 participants, 1248 (17%) had a long term sickness absence (LTSA) episode (>16 days) over the 1 year follow-up. The main study population characteristics at baseline are displayed in **Table 1**. The mean age was 43 years and women (58%) comprised of a larger proportion of the population. Univariate analysis showed significant associations between sickness absence and being female, being divorced, separated or widowed, lower education, lower income, higher alcohol intake, smoking, and lack of physical activity.

**Table 1:** Baseline characteristics of study population and their association with an episode of long-term sickness absence.

	No SA		SA		p-value^a^
	N (%)	Mean (SD)	N (%)	Mean (SD)	
**Gender**					
Male	2688 (44)		380 (30)		
Female	3410 (56)		868 (70)		**<0.001**
**Age**	–	43.17 (1.54)		43.23 (1.54)	0.216
**Marital status**					
Unmarried	722 (11.8)		151 (12.1)		
Married	4664 (76.5)		854 (68.4)		
Widowed/divorced/separated	712 (11.7)		243 (19.5)		**<0.001**
**Number of biological children**					
0–2	3605 (60.1)		742 (60.3)		
3 or more	2391 (39.9)		488 (39.7)		0.895
**Education**					
Compulsory school	963 (15.8)		278 (22.3)		
High school	2736 (44.9)		604 (48.4)		
1–3 years at university/college	1235 (20.25)		210 (16.8)		
4+ years at university/college	1164 (19.1)		156 (12.5)		**<0.001**
**Income after tax**	–	189302.7 (110245)	–	171922 (67981)	**0.0001**
**BMI**		25.21 (3.7)		25.21 (3.7)	0.194
Normal (<25)	3208 (52.7)		666 (53.4)		
Overweight (25–30)	2271 (37.3)		444 (35.6)		
Obese (≥30)	614 (10.1)		138 (11.1)		0.391
**Alcohol consumption**					
Abstainer	1618 (26.6)		395 (31.8)		
Normal	4167 (68.4)		803 (64.6)		
High	303 (5)		46 (3.7)		**<0.001**
**Smoking**					
No	3941 (66.9)		665 (55.3)		
Yes	1947 (33.1)		537 (44.7)		**<0.001**
**Physical Activity (Hard exercise)**					
No	1574 (26.5)		389 (32.2)		
Rare	1691 (28.5)		308 (25.5)		
Some	1819 (30.7)		354 (29.3)		
Frequent	850 (14.3)		158 (13.1)		**0.001**

a p-value obtained using independent t-test (BMI, continuous), Kruskal-Wallis test (income, age), Fisher’s exact test (marriage), and Chi-squared test for all other variables.

As expected, higher job strain was associated with more somatic symptoms, higher HADS scores and lower SF-12 scores (all p = 0.0001, data not shown). The number of chronic somatic diseases (p = 0.464) and pharmacological diagnoses (p = 0.464) did not differ significantly across the job strain categories. The univariate associations between mental and physical health indicators and LTSA were significant (p-value <0.001) and in the expected direction (data not shown).

**Figure 1:**
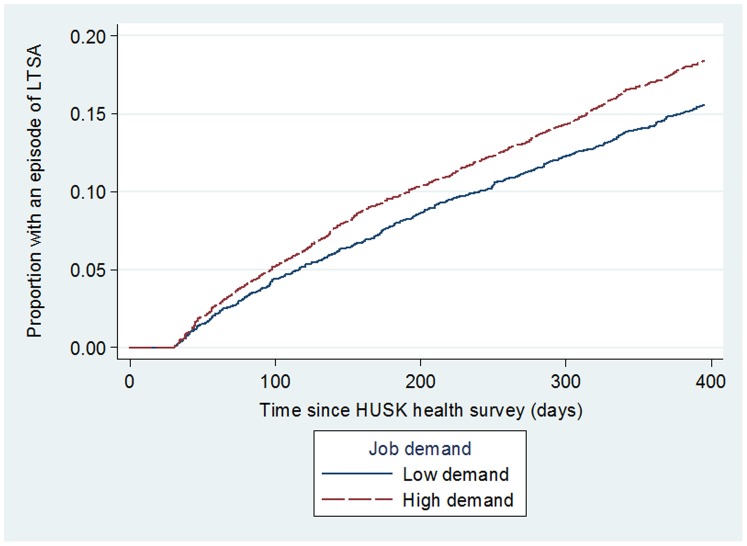
Unadjusted risk of participants having an episode of long term sickness absence (LTSA) by job demand.

**Figure 2:**
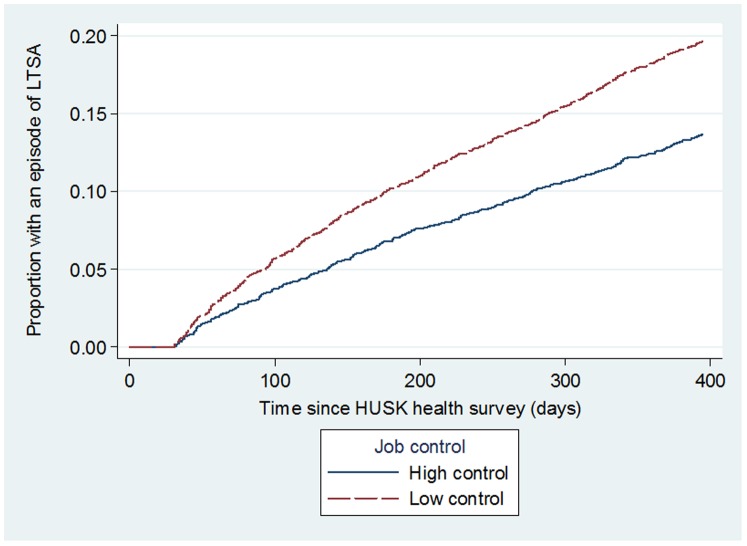
Unadjusted risk of participants having an episode of long term sickness absence (LTSA) by job control.

**Figure 3:**
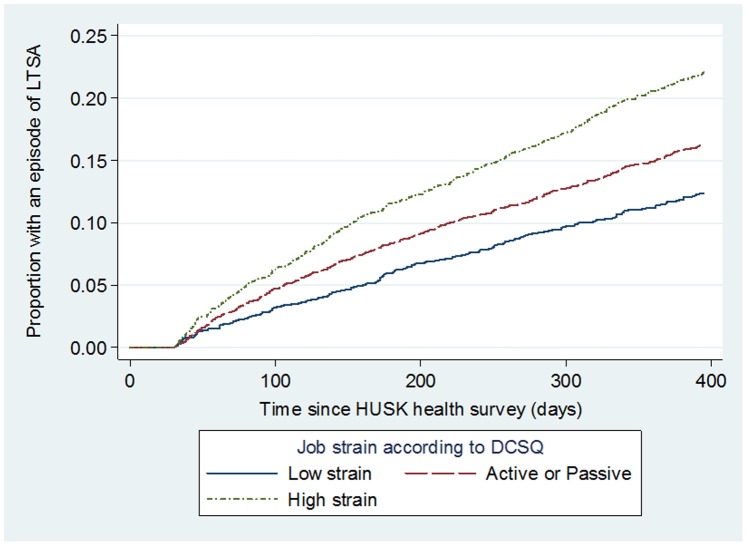
Unadjusted risk of participants having an episode of long term sickness absence (LTSA) by job strain.

High job demand **(** **Figure 1** **)** and low job control **(** **Figure 2** **)** were univariately associated with higher risk of LTSA (p = 0.001 and p<0.001 respectively). Similarly, **Figure 3** shows that the proportion of participants with a LTSA episode was highest for those with high job strain (p<0.001). **Table 2** shows that compared with those in low strain jobs, employees in active or passive work (intermediate strain jobs) and high strain jobs were at increased the risk of LTSA, with HRs of 1.34 (95% CI = 1.14–1.59) and 1.89 (95% CI = 1.58–2.25) respectively (model 1). Adjusting for potential confounders (model 2) attenuated the HRs to 1.27 (95% CI = 1.07–1.51) and 1.64 (95% CI = 1.36–1.98), but the p-value for linear trend remained significant (p<0.001). No significant effect modifications by age (continuous) (p = 0.729), gender (p = 0.628), or number of biological children (p = 0.903) were detected. Population attributable fraction (PAF) estimates revealed that incidences of LTSA would be reduced by 6.76% (95% CI: 4.43–9.04%) had there been no exposure to high job strain in the working population.

**Table 2:** Associations between job strain and sickness absence using multivariate Cox proportional hazards regression models.

	n	Hazard Ratios (95% CI)		
		Model 1	Model 2	Model 3
		(unadjusted)	(+ confounders)	(+ physical and mental health)
**Level of Job strain (DCSQ)**				
Low strain	1444 (20)	1.0 (Ref)	1.0 (Ref)	1.0 (Ref)
Active or Passive	4026 (55)	1.34 (1.14–1.59)	1.27 (1.07–1.51)	1.19 (1.00–1.43)
High strain	1876 (25)	1.89 (1.58–2.25)	1.64 (1.36–1.98)	1.30 (1.07–1.59)
p-value for linear trend		**<0.001**	**<0.001**	**0.011**
**Sociodemographic factors**				
Gender				
* Male*		–	1.0 (Ref)	1.0 (Ref)
* Female*		–	1.59 (1.38–1.83)	1.40 (1.20–1.62)
Age		–	1.02 (0.98–1.06)	1.00 (0.96–1.04)
Education		–	0.88 (0.82–0.94)	0.89 (0.83–0.96)
Income		–	1.00 (0.99–1.00)	1.00 (0.99–1.00)
Number of biological children				
* 0–2*		–	1.0 (Ref)	1.0 (Ref)
* 3 or more*		–	1.09 (0.96–1.23)	1.13 (0.99–1.29)
Marital status				
* Unmarried*		–	1.0 (Ref)	1.0 (Ref)
* Married*		–	0.78 (0.64–0.94)	0.87 (0.71–1.06)
* Widowed/divorced/separated*		–	1.29 (1.03–1.60)	1.23 (0.97–1.55)
**Lifestyle factors**				
Smoking				
* No*		–	1.0 (Ref)	1.0 (Ref)
* Yes*		–	1.40 (1.23–1.58)	1.30 (1.14–1.48)
Alcohol Consumption		–	0.87 (0.77–0.98)	0.89 (0.79–1.01)
Physical Activity		–	0.99 (0.93–1.05)	1.05 (0.98–1.11)
BMI		–	1.08 (0.99–1.17)	0.99 (0.91–1.09)
**Physical Health**				
Somatic Symptoms		–	–	1.01 (1.00–1.02)
Somatic Diseases		–	–	1.16 (0.97–1.40)
Pharmacological Diagnosis		–	–	1.27 (1.09–1.47)
SF-12 PCS		–	–	0.95 (0.95–1.01)
**Mental health**				
HADS-A		–	–	0.98 (0.95–1.01)
HADS-D		–	–	0.97 (0.95–1.00)
SF-12 MCS		–	–	0.97 (0.96–0.98)

Model 1 - Unadjusted/Crude.

Model 2 - Adjusted for sociodemographic factors, and lifestyle factors.

Model 3 - Adjusted for sociodemographic factors, BMI, lifestyle factors, physical health and mental health.

Finally, the potential role of physical and mental health symptoms as mediators in the relationship between job strain and LTSA was assessed. Physical health and mental health individually attenuated the association between high job strain and sickness absence, although not substantially (data not shown). In the fully adjusted model (model 3), which controlled for physical and mental health measures together in addition to confounding factors, the association between high strain jobs and sickness absence was attenuated but remained significant (HR = 1.30; 95% CI = 1.07–1.59). For intermediate strain jobs, the association was no longer significant after controlling for health measures (HR = 1.19; 95% CI = 1.00–1.43). The same analyses were repeated for active and passive job strain separately and the results for the two categories did not differ significantly (data not shown).

## Discussion

In this prospective study we found a significant association between job strain and long term sickness absence (LTSA); participants with higher job strain were at increased risk of taking LTSA over a one year follow up period. In addition, population attributable fraction estimates suggest that 1 in 15 of LTSA episodes could have been prevented had there been no exposure to high job strain. Our results also showed that impairment in physical and mental health only partly explained the relationship between job strain and LTSA.

Our findings are in line with those reported by several large prospective European studies. In a Finnish working population, it was reported that there was a 17% (women) and 41% (men) increased risk for LTSA among employees with high job strain [39]. In the French Gazel cohort, researchers found that exposure to the highest level of work and family demands, combined, lead to a 3.55-fold and 6.58-fold increase in risk of psychiatric sickness absence for male and female employees, respectively [24]. However, the current results contrasts with those from the Whitehall Study [40], which found that job strain was no longer a predictor of LTSA, following adjustments for potential confounders. The discrepancies may be explained by differences in the baseline population, in terms of age range (aged 30–55) and occupation (nonindustrial civil servants), as well as differences in the qualifying length of sickness absence.

To the best of our knowledge this is the first study based on a middle-age, disability free, working population to show that employees reporting high job strain are at increased risk of LTSA. This is important as LTSA accounts for one third of the days off work and 75% of all absence costs [19]. Our study has also clarified that both job demands and job control are associated with LTSA.

The most commonly proposed explanation for the association between job strain and sickness absence is that job strain causes physical and mental illness that results in the need to take SA [40]. However, it has been recognized that sickness absence is a complex and multifactorial phenomenon [41] that is not a direct function of illness severity [15], [19]. The data used in our study enabled us to determine the extent to which these health indicators could explain the link between job strain and LTSA. Our results suggest that mental and physical health could only partially account for this association, and thus other pathways are likely to be important.

Past studies have partially accounted for health measures in their analyses, adjusting for either physical [39] or mental health [24], and similarly did not find significant attenuations in the job strain and sickness absence association. One study that controlled for a more complete set of health measures reported that job strain remained a significant predictor of disability pension following the adjustments [42]. To the best of our knowledge, no previous study has directly examined both physical and mental health as constituents on the causal pathway between job strain and sickness absence. One possible reason for the failure of mental and physical health to explain the link between job strain and sickness absence may be due to the lack of distinction between the varying lengths of the LTSA episode. Studies have suggested that compared to psychosocial factors, ill health is a stronger predictor of longer absence spells [43]. Another possible explanation for our findings is that participants’ health conditions changed during the follow-up, which may have prevented the complete adjustment for health.

Researchers have started investigating other non-health related factors that may be involved in the network of pathways connecting job strain to sickness absence. Studies have shown that factors pertaining to the work environment, such as job dissatisfaction, and organizational factors such as management style and absence culture [35], [44] are likely to be implicated in sickness absence behavior. In addition, personal or individual factors, including occupational grade [23], perception of health and vulnerability [45], attitudes towards work [46], and personality [35], may alter the tendency for an employee to rate their job as high strain and take LTSA. More upstream factors including early childhood experiences and temperament, may also be important in the development of many of these personal factors and have in themselves been postulated to affect LTSA during adulthood [16], [17], [47]. A recent study [48] performed on Norwegian Armed Forces employees reported that hardiness, a trait that develops early in life and stays stable over time, interacts with job strain to affect sickness absence behavior. Furthermore, non-work factors including social relations and work-family demands [24], [49] have been demonstrated to predict all-cause sickness absence; these factors could also have affected an employee’s perception of job stress and may be the true drivers of illness and sickness absence. Taken together with these emerging findings, our results suggest that simplistic models of job strain leading to ill health and sickness absence need to be modified to account for the complex and multi-factorial nature of illness and sickness absence behavior. High job strain is associated with increased physical and mental ill health and higher levels of LTSA. However, each step on this pathway is not a simple progression, but appears to be influenced by a range of other individual and organizational factors. Given this extensive literature demonstrating the influence of individual and other psychosocial factors on sickness absence, it is perhaps unsurprising that our study found that health factors could only moderately explain LTSA.

A methodological strength of this study is its prospective design, which enables the establishment of a temporal sequence and also reduction of response bias. Furthermore, the sickness absence data retrieved from official Norwegian registries, is highly accurate and complete, and therefore excludes the possibility of recall and measurement bias. The HUSK study collected a range of socio-demographic and lifestyle variables, which enabled our study to account for the impact of many potential confounders. Also, the baseline measures of mental and physical health were reliable and obtained using well-validated psychometric scales.

However, this study is not without limitations. First, our study population is restricted to middle-aged Norwegian employees who receive generous sickness benefits, which may limit the generalizability of the results. Due to differences in policies and benefit entitlements, these results may not be generalizable to employees of other countries with much lower rates of sickness absence. However, the study population is comparable to the general working population in that the proportion of sickness absences in those aged 40–49 (6.2–6.3%) is similar to that among the total working population (aged 16–69, 6.5%) [50]. Secondly, although the participation rate was acceptable, previous studies using the HUSK dataset have reported higher non-participation rates amongst individuals with mental disorders [51]. Third, the follow-up period of one year was relatively short, and could have led to an underestimation of job strain effects on LTSA. Fourthly, finding an adequate measure for physical health was difficult. To address this issue we used four different physical health outcomes, but it is likely that certain physical health outcomes overlap, resulting in an overestimation of physical health influences on LTSA. On the other hand, the use of an unweighted cumulative score for chronic somatic diseases assumes equal severity of all somatic conditions, which could have resulted in an underestimation of the effect. Similarly, the different psychometric health measures may have overlapped or did not cover all possible illnesses, thereby reducing our ability to accurately estimate their mediation effects. The apparent inability of health measures to explain the relationship between job strain and LTSA could be due to the measurement errors and the crudeness of the health measures used. Additionally, health measures were only collected at baseline; therefore we could not capture changes in health status throughout the follow-up. Given the dynamic nature of health it is possible that an individual with good health at baseline could have developed new health problems during the follow up period, which could influence the risk of LTSA. Fifth, the use of official records of SA restricted the analysis to longer spells of sickness absence (>16 days). Also, despite growing recognition for the importance of individual and non-health factors including family demands, personality, organization, and occupational grade, in explaining sick leave, these variables were not collected in the HUSK study and could not be assessed. Finally, health data was based on self-reports that are prone to reporting and recall bias, and consequently misclassification. Future research may focus on work unit-aggregated job strain measures [39], [42] to reduce biases introduced by self-rated job strain, and determine whether health measures can explain the associations with sickness absence.

In conclusion, the present study shows that employees who report high levels of job strain are at increased risk of LTSA. The use of highly accurate sickness absence records coupled with adjustments for a broad range of potential confounders enabled us to corroborate existing literature on the utility of the job strain model in assessing how the psychosocial work environment can influence health and occupational outcomes. Our results suggest that one in fifteen episodes of LTSA could have been avoided if high levels of job strain were eliminated. However, our results also caution against risk management approaches and models which assume direct causal links between self-reported stress, ill health and sickness absence. The impact of job strain on future LTSA could not be fully explained by impaired health at baseline, which suggests that factors besides ill health may be important and affect the employee’s threshold for taking sickness absence.
